# 

**Published:** 1899-12-02

**Authors:** 


### NOTICE TO CORRESPONDENTS.

All MS., Letters, Books for Review, and other matters intended for the Editor should be addressed The Editor, "The Hospital," The Hospital Building, 28 & 29, Southampton Street, Strand, W.C.

The Editor cannot undertake to return rejected MS., even when accompanied by stamped directed envelope.

**Notice to Advertisers and Subscribers.**

All Advertisements, Orders for Copies of The Hospital, and Business Communications should be addressed to **THE MANAGER (Not to the Editor)**, The Hospital, The Hospital Building, 28 & 29, Southampton Street, Strand, London, W.C.

The Cost of Subscription, post free, is as follows:—Per week, 2½d.; per quarter, 2s. 8d.; per half-year, 5s. 3d.; per annum, 10s. 6d. Foreign:—Per annum, 15s. 2d., payable, in all cases, in advance.

**NOTICE.—Vol. XXVI. of "The Hospital" (April, 1899, to September, 1899), in handsome cloth binding, is now on sale at the Office of this Journal, price 7s. 6d. Cases for binding the Numbers contained in this Volume 1s. 6d. Index to Vol. XXVI., 2½d., post free.**

**The Monthly Part for December is now ready. Price 6d., by post 8d.**

### BOOKS RECEIVED.

T. O. Todd, Sunderland.

"Life in a Provincial Workhouse Hospital." By Annie S. Pruett.

John Bale, Sons, and Danielsson.

"On the Prevention of Eye Accidents Occurring in Trade." By Simeon Snell, F.R.C.S.Ed.

"The Medical Digest Appendix, including the Years 1891 to March, 1899." By Richard Neale, M.D.Lond.

"British Sanatoria for the Open-Air Treatment of Tuberculosis."

John Wright and Co.

"The Medical Annual Synoptical Index to Remedies and Diseases, 1887 to 1898."

Hutchinson and Co.

"In the Years that Came After." By Mrs. Fred Reynolds.

**Periodicals and Pamphlets.**—Sunday at Home—Boy's Own Paper—Medical Press—Medical Record—Scalpel—Nursing Notes—Literary Digest—Guy's Hospital Gazette—Health—Educational Review—Charity Organisation Review—Leisure Hour—Asylum News—Hospital Sanatorium Record.

### The Student of Medicine.

### APPOINTMENTS.

E. M. Barker, M.A., M.B.&B.C.Cantab., L.R.C.P., M.R.C.S., appointed Honorary Medical Officer to the Convalescent Home of the Chelsea Hospital for Women at St. Leonard's-on Sea. Sir H. Beevor, Bart., M.D., appointed Physician at King's College Hospital. W. A. Carte, M.D., M.Ch.Univ.Dub., appointed Senior Assistant Medical Officer to the Lagos Railway, West Africa. C. C. Douglas, M.B., B.Sc.Edin., appointed Professor of Medical Jurisprudence in Anderson's College Medical School, Glasgow. F. C. Fischer, M.D., F.R.C.S Edin., appointed Professor of Ophthalmology to the School of Medicine, Cairo. J. F. R. Gairdner, M.B., C.M.Glasg., appointed Extra Dispensary Surgeon, Royal Hospital for Sick Children, Glasgow. R. Gibson, M.B., Ch.B.Edin., appointed House Physician to the Hospital for Women, Soho Square, W. J. G. Gray, M.B., C.M.Glasg., appointed Extra Dispensary Physician, Royal Hospital for Sick Children, Glasgow. R. L. Grosvenor, B.A.Cantab., M.R.C.S., L.R C.P., appointed Clinical Assistant, Chelsea Hospital for Women. G. H. S. Hillyar, L.R.C.P., L.R.C S.Edin., appointed Medical Officer for the Much Wenlock District of the Madeley Union. Miss B. Knowles, M.B., B.S.Lond., appointed Assistant Medical Officer to the Waterloo Road Workhouse of the Bethnal Green Union. E. S. Littlejohn, B.A.Syd., M.D., C.M.Edin., appointed Honorary Medical Officer to Outpatients' Department, Sydney Hospital for Sick Children. H. J. D. Mackay, L.R.C.P.E., L.R.C.S.E., L.F.P.&S., appointed Medical Superintendent of Aston Hall, Warwickshire. J. F. McLean, M.R.C.S., L.R.C.P.Lond., appointed Superintendent Surgeon to the British Seamen's Hospital, Constantinople. J. R. MacMahon, M.B., C.M., appointed Clinical Assistant to the Chelsea Hospital for Women. G. N. Meachen, M.B.Lond., M.R C S., L.R.C.P., appointed House Surgeon to the Tottenham Hospital, N. J. Molineux, M.D.St.And., M.R.C.S.Eng., appointed Medical Officer of Health to the Hessle Urban District. G. Norman, M.B., appointed Medical Officer for the Buckhurst Hill District of the Epping Union. H. Stuart, M.R.C.S., L.R.C.P., appointed Assistant Resident Medical Officer to the London Temperance Hospital. W. Wardman, L.R.C.P., L.R.C.S.Edin., appointed Medical Officer for the Third District of the Manchester Union. A. A. Young, M.A., M.B., C.M.Glasg., appointed Extra Dispensary Surgeon, Royal Hospital for Sick Children, Glasgow.

King's College Hospital.—The following appointments have been made: House Physicians—(Senior) G. A. Roberts, M.R C.S., L.R.C.P.; (Junior) W. H. McMullen, M.R.C.S., L.R.C.P.; (Assistant) F. A. Hadley, M.R C.S., L.R.C.P. House Surgeons: S. M. Mayon, M.R.C S., L.R C.P.; H. B. Stratford, M.R.C.S., L.R.C.P.; P. Vosper, M.R.C.S., L.R.C P. House Accoucheurs: C. E. Fenn, M.R.C.S., L.R C.P.; (Assistant) J. M. Twentyman, M.R.C.S., L.R.C.P.

### NOW READY,

A REPRINT OF

### A NURSING HANDBOOK

TOGETHER WITH S. MAW, SON, AND THOMPSON'S

### COMPLETE CATALOGUE OF NURSING REQUISITES.

The above work is now republished at a cost of 1s. per copy. Messrs. S. M., S., and T. have been compelled to make this charge on account of the extreme popularity of the work and the great expense incurred in its production.

***Post Free on receipt of 1s.***

### S. MAW, SON, and THOMPSON,

7 to 12, ALDERSGATE STREET, LONDON, E.C.

